# Best practice workplace HIV/AIDS programmes in South Africa: A review of case studies and lessons learned

**DOI:** 10.4102/phcfm.v1i1.30

**Published:** 2009-07-21

**Authors:** Geoffrey K.G. Setswe

**Affiliations:** 1 Human Sciences Research Council, Pretoria, South Africa

**Keywords:** best practice, workplace, HIV/AIDS, programmes, prevention

## Abstract

**Background:**

A group of experts attending a tripartite interregional meeting on best practices in HIV/AIDS workplace policies and programmes organised by the International Labour Organisation (ILO) in Geneva, Switzerland, identified 34 best practice workplace HIV programmes from across the world.

**Method:**

The ten criteria that were used for reviewing best practice workplace HIV/AIDS programmes in South Africa include acceptability, accessibility, ethical soundness, perceived impact, relevance, appropriateness, innovativeness, efficiency, sustainability and replicability.

**Results:**

More than one-third (35.3%) of the 34 best practice workplace interventions identified were found in businesses and industries in South Africa. This constitutes a significant and encouraging effort to deal with HIV/AIDS in the workplace. Approximately 16.7% of the best practice workplace HIV/AIDS interventions focused on policy and legal frameworks, 50% of these interventions focused on prevention, 16.7% provided links beyond the workplace and a further 16.7% were interventions that focused on knowledge and evidence. A third (33.3%) of practices were found in the mining industry, 16.7% in the motor industry, 16.7% from workers’ unions, and the rest (33.3%) were found in a sugar company, an electricity supply company, a pharmaceutical company and the ministry of Public Service and Administration.

**Conclusion:**

It is encouraging that over one-third of all best practice workplace HIV interventions identified by the ILO experts were found in South Africa. The majority of these policies and programmes were focused on HIV prevention.

## INTRODUcTION

Within the past few years increased concern has been expressed about the impact of the HIV epidemic on the achievement of sustainable development in southern Africa. At least 23 million workers aged 15 to 49 years are infected with the HI virus^1^ and over 90% of those living with HIV are adults in the prime of their lives. Workplace HIV/AIDS programmes are rarely evaluated to determine if they provide the best possible prevention, treatment, care or support for employees.

Workplace programmes refer to a range of company-based interventions including the development and implementation of an HIV/AIDS policy, voluntary counselling and testing (VCT), and provision of antiretroviral therapy (ART).^2^

The impact of HIV/AIDS on employees within workplaces has certain features in common. When HIV/ AIDS morbidity begins, sick leave and other forms of absenteeism increase, the overall productivity of the workforce declines due to employee illness, the overall labour costs increase, overtime and contractors’ wages increase to compensate for absenteeism, and the increased use of medical aid benefits causes premiums to rise. A large number of workplace HIV/AIDS programmes which implement a variety of interventions are rarely evaluated to determine their effectiveness.^3^

A best practice must at least demonstrate evidence of success, contribute to the organisation's mission or programme goals, and have the potential to be replicated or adapted to other settings.^4^ A best practice must be innovative, sustainable, cost-effective, ethically sound and/or superior to all other approaches.^5^ Deciding on and testing what is ‘best’ is not easy. A best practice must actually be tested in the workplace. It is most appropriately identified at the level at which it is practised and in consultation with as many of the workers and managers concerned as possible because the people directly involved are ideally placed to determine what actually works.^6^

A group of experts attending a tripartite interregional meeting on best practices in HIV/AIDS workplace policies and programmes organised by the International Labour Organization (ILO)^7^ in Geneva, Switzerland, in December 2003 identified 34 best practice workplace HIV programmes from across the world. The purpose of this paper is to identify and review best practice workplace HIV/AIDS interventions that are based in South Africa.

## METHOD

This study reviewed and synthesised the ILO report on the best practice workplace HIV/AIDS policies and programmes from across the world to identify and describe best practice workplace HIV/AIDS programmes from South Africa.^8^ The 10 criteria that were used for identifying best practice workplace HIV/AIDS programmes appear in Table 1.

**TABLE 1:** Criteria for identifying best practice workplace HIV/AIDS programmes

CRITERION	BRIEF DESCRIPTION
Acceptability	Favourably regarded by the beneficiaries, the authorities and professional bodies. Reflects generally accepted values and the principles in prevention, treatment, care or support of OVC.
Accessibility	Includes a commitment to involve all social partners and experts selected by them when necessary. Optimally available within reach when needed.
Ethical soundness	The project is ethical and meets universal standards of compassion, tolerance, respect, confidentiality, empowerment and participation. Sensitive to workers’ rights, conforms to ethical standards and does not break principles of social and professional conduct.
Perceived impact	Anticipated positive change in magnitude of problem. Reflects best available evidence in implementation of workplace HIV/AIDS interventions. Incorporates systematic monitoring and evaluation of its impact.
Relevance	Is relevant and tackles the problems faced by workers. How closely a workplace intervention is focused on the HIV/ AIDS response in the context of the society in which it is implemented.
Appropriateness	Is appropriate to the situation, country and culture. Does not go against social, political norms accepted and practiced locally or by the intended beneficiaries.
Innovativeness	Demonstrates creativity and breaks new ground. Has been documented and shown to be effective in more than one setting or situation.
Efficiency	Demonstrated capacity to produce desired results with a minimum expenditure of energy, time or resources. Is affordable and adds value.
Sustainability	Ability of the intervention to continue effectively and to maintain levels of achievements over the medium to long term. Is sustainable in terms of structures, capacity and funding to continue working over the long term without outside support.
Replicability	The essential elements i.e. technology, resources and organisation of the intervention can be easily applied elsewhere in response to a similar problem and produce similar results.

Criteria used for identifying and reviewing best practice workplace HIV/AIDS programmes include:

1. **Acceptability:** The programme is favourably regarded by the employees, employers and other stakeholders. It reflects generally accepted values and principles in prevention, treatment, care or support of employees in the workplace.
2. **Accessibility:** The project includes a commitment to involve all HIV/AIDS stakeholders in the workplace and is optimally available, within reach, when needed.
3. **Ethical soundness:** The project is ethical and meets universal standards of compassion, tolerance, respect, confidentiality, empowerment and participation. It is sensitive to workers’ rights, conforms to ethical standards and does not undermine principles of social and professional conduct.
4. **Perceived impact:** The project reflects best available evidence in the implementation of workplace HIV/AIDS interventions and incorporates systematic monitoring and evaluation of impact.
5. **Relevance:** The workplace intervention is relevant and tackles the problems faced by workers and is focused closely on the HIV/AIDS response in the societal context in which it is implemented.
6. **Appropriateness:** The project is appropriate to the situation, workplace and culture and does not go against social and political norms accepted and practised locally or by the intended beneficiaries.
7. **Innovativeness:** The project demonstrates creativity, breaks new ground and has been documented and shown to be effective in more than one setting or situation.
8. **Efficiency:** The programme is affordable and adds value, and has demonstrated capacity to produce desired results with a minimum expenditure of energy, time or resources.
9. **Sustainability:** The HIV/AIDS programme has the ability to continue effectively and to maintain achievement levels over the medium to long term. The programme is sustainable in terms of structures, capacity and funding, to continue working over the long term without external support.
10. **Replicability:** The essential elements such as technology, resources and organisation of the programme can be easily applied elsewhere in response to a similar problem and produce similar results.^1^

## RESULTS

Table 2 shows a summary of best practice workplace HIV/AIDS policies and programmes in South Africa. More than one-third (35.3%) of the 34 best practice workplace interventions were found in the business and industry sectors of South Africa. This constitutes a significant and encouraging effort to deal with HIV/AIDS in the workplace.

**TABLE 2:** Summary of best practice workplace HIV/AIDS policies and programmes in South Africa

INTERVENTION AREA	INTERVENTION	WORKPLACE AIDS PROGRAMME	TYPE OF WORKPLACE
Policy and legal frameworks	Sectoral agreement between multinational and trade unions	Anglo Gold South Africa, and the NUM, MWU-S, NETU, SAEWA, UASA	Gold mining
Workplace policies and programmes: prevention	Tackling stigma and discrimination– building trust between the partners	Illovo Sugar	Sugar industry
	Behaviour change – personal risk assessment and change strategies	BMW South Africa	Motor industry
	Behaviour change – self-help groups and partnerships for change	South African Clothing and Textile Workers Union (SACTWU)	Workers’ union
	Behaviour change – safe use of needles at work	Democratic Nursing Organization of South Africa (DENOSA)	Nurses’ union
	Stigma and discrimination - confidentiality for prevention and care	De Beers	Diamond mining
	Increasing uptake of VCT	Eskom	Electricity supply company
	Treatment – responding to opportunistic infections through partnership	Pfizer – South Africa Alliance	Pharmaceutical industry
Links beyond the formal workplace	Managing the transition from formal to informal	Placer Dome Western Areas Joint Venture	Gold mining
	The community – treatment for STIs and social outreach	The Lesedi project	Gold mining
Knowledge and evidence	Situation analysis – understanding what is needed	Daimler Chrysler South Africa (DCSA)	Motor industry
	Monitoring and feedback	Ministry for Public Service and Administration	Public service

About 16.7% of the best practice workplace HIV/AIDS interventions focused on policy and legal frameworks, (50%) of these interventions focused on prevention, 16.7% provided links beyond the workplace and a further 16.7% were interventions that focused on knowledge and evidence.

A third (33.3%) of practices were found in the mining industry, 16.7% in the motor industry, 16.7% from workers’ unions and the remaining 33.3% were found in a sugar company, an electricity supply company, a pharmaceutical company and the ministry of Public Service and Administration.

### Sectoral agreement between multinationals and trade unions

Large multinational corporations have often been at the forefront of workplace responses to HIV/AIDS to deal with the economic threat posed by the pandemic. Trade unions, particularly in South Africa, have been all too aware of the impact of HIV/AIDS on their members. This case study illustrates the extent to which these groups can define common ground and work together to establish a framework which is in the common interest of all.

**AngloGold and five unions**

(National Union of Mineworkers (NUM), Mine Workers Union - Solidarity (MWU-S), National Employees’ Trade Union (NETU), South African Equity Workers’ Association (SAEWA), United Association of South Africa (UASA):

AngloGold has reached a comprehensive collective agreement on a framework for action with five unions covering management of HIV/AIDS at work. It builds on best practice, draws on international codes, and creates an ongoing partnership. Placing the issue in the collective bargaining sphere is appropriate in some settings but not in others, and is not the only way of formulating workplace policies.

#### Lessons learned

The collaboration between AngloGold and the five unions has assisted all partners to explicitly define the aims, rights and responsibilities of different parties and has allowed partners to be clear about where they stand in relation to each other and their commitments. It has been found helpful to include specific references to best practice and quality standards as it allows for effective monitoring of progress and review of ethical standards.

The case study has shown that integrating commitments on HIV/AIDS in collective bargaining does not mean that funds for prevention and care should be seen as interchangeable or ‘in competition’ with funds for pay or other remuneration or benefits. Extending partnerships between social partners to include non-governmental organisations (NGOs) and technical experts strengthens credibility and can be the key in extending initiatives to the community.

### Tackling stigma and discrimination by building trust between partners

The stigma and discrimination surrounding HIV/AIDS is not only contrary to human rights but also represents a major obstacle to successful workplace programmes. The fear of rejection, shame and discrimination undermines efforts to promote behaviour change, inhibits people from using VCT services or obtaining treatment, and prevents their seeking care for opportunistic infections. Non-discrimination policies help to create a non-judgemental and supportive culture.

#### Lessons learned

In tackling stigma and discrimination in the workplace, it is essential to involve stakeholders and particularly unions as early as possible to build trust. In this case study, a committee was formed which actively involved all parties. This sends a clear signal that the initiatives undertaken are trustworthy and intended to support all workers. It is not necessary to create new structures where suitable vehicles already exist (e.g. a health and safety committee), as long as commitment to the new agenda is genuine.

**Illovo Sugar**

Illovo Sugar has a combined prevention and care programme which has adopted a multi-stakeholder, multi-disciplinary approach. The company worked with unions, management, occupational health services and medical and academic experts to ensure that the programme was properly embedded in the organisational culture and could inspire trust. It provides access to condoms and educational activities, as well as care and support.

#### Lessons learned

The lesson learned in this case study is that making explicit commitments to confidentiality from the outset by the committee is important. Again, mass meetings that directly involve workers supplement the union's role and provide an additional route for the dissemination of information and a public statement of commitment and openness.

### Behaviour change: Personal risk assessment and change strategies

HIV transmission is preventable but this depends not just on an understanding of the processes of HIV transmission but also on individual actions taken to prevent infection. There are many reasons why people do not take action to protect themselves and their families. The scope for individual action is often constrained by social and other conditions. Workplace initiatives can support and empower individuals to make changes. Participatory education programmes and practical supportive measures can help people assess their risk, become aware of their attitudes, and change their behaviour.

**Bavarian Motor Works (BMW) South Africa**

BMW has a comprehensive programme for tackling HIV/AIDS, based on a policy agreed upon with the unions and supported at the highest level of management. It involves a range of elements, including the provision of Highly Active Antiretroviral Treatment (HAART). It also places considerable emphasis on prevention using a comprehensive communications strategy and various awareness-raising formats (workshops, events, theatre, etc.).

BMW facilitates personal risk assessment for staff, particularly women, which empowers them by allowing them to review their own behaviour and exposure to risk, and to identify the elements they may want to change, as well as the impediments to and enablers of change.

#### Lessons learned

The lesson learned in this case study is that working in women- or men-only groups helps individuals to express themselves with confidence and learn from each other. To get the most benefit from personal risk assessments, implementers should include practical sessions addressing the reality of risk and issues such as negotiating condom use and discussing HIV status with a partner.

It is essential to use tools such as role-play, which allow people to ‘practise’ how to handle difficult situations, and to allow people to rehearse the ways in which they would respond to sensitive situations. Behaviour change programmes should make use of peer educators to facilitate risk assessment but they need to be supported, ideally through regular support sessions and through additional training.

Trust is crucial to the success of behaviour change programmes and the prioritisation of privacy and confidentiality builds confidence. Providing HAART creates a significant incentive for VCT uptake and sustaining safer sexual behaviour.

### Behaviour change through self-help groups and partnerships for change

Providing knowledge to employees about HIV/AIDS does not translate to change in behaviour. The approach used in this best practice intervention is to change behaviour through self-help groups and the creation and support of partnerships for change.

**South African Clothing and Textile Workers’ Union (SACTWU)**

SACTWU has its own HIV policy and has trained shop stewards to be aware of the issues and to implement policy appropriately. It provides ARV treatment to prevent mother-to-child transmission (MTCT) and is developing a policy for home care and orphan support. It works with employers and is actively involved in a series of partnerships with not-for-profit and non-governmental organisations. It collaborates with education specialists in designing and delivering training with health NGOs to see that TB is addressed through the DOTS initiative, and supports the Treatment Action Campaign, which is lobbying for national ARV provision. Its support to community, workplace and campaign groups facilitates self-help initiatives by People Living with HIV/AIDS (PLWHA), which empowers individuals and promotes behaviour change.

#### Lessons learned

The lesson learned from this programme is that mobilising people affected by HIV/AIDS through self-help groups is effective in encouraging behaviour change. Self-help groups are often most effective when focused on issues in which members have a direct stake and which play to their strengths. Incorporating self-help groups into multi-sectoral coalitions ensures that partnerships are informed by stakeholders’ views and that what is involved in changing behaviour is understood. It is important to create a forum for sharing the major objectives to ensure that all players can see how they fit in and can pool relevant information.

### Behaviour change through safe use of needles at work

Much of the focus on behaviour change has been on safe sex and harm-reduction in intravenous drug use but it is also an issue of direct relevance to safety in the workplace. The ILO code of practice calls for a healthy work environment and includes in this a supportive setting in terms of physical and mental health, and adaptation of work to the needs of PLWHA. It also demands that Universal Precautions are observed in workplaces where workers come into contact with human blood and body fluids.

**The Democratic Nursing Organisation of South Africa (DENOSA)**

In seeking to promote a healthy working environment, the DENOSA has highlighted the employers’ responsibilities for ensuring that people change the way they work in contact with blood, and has actively sought to promote change around the handling of sharp instruments. It has also made the links with prevention and nondiscrimination.

#### Lessons learned

DENOSA found that introducing messages about safe working practices as early as possible in training helps change cultures as well as the habits of nursing professionals. This case study has also shown that workers need time and resources if they are to change established practices and follow new safety guidelines.

Any training or demands for behaviour change which touch on the risk of HIV/AIDS may provoke unexpected resistance because of the stigma associated with the disease, even amongst health workers. Any measures to encourage uptake of universal precautions is an opportunity to widen discussion and tackle broader prevention issues.

### Maintaining confidentiality to avoid stigma and discrimination

Confidentiality policies are widespread, and in line with the ILO code they tend to protect job applicants and workers as well as all the personal data relating to a worker's HIV status. These issues are more complex in an informal setting, where self-employment and casual employment are commonplace: records will rarely be held, but personal confidences may be made and should still be bound by rules of confidentiality. The most useful learning points now tend to revolve around measures for ensuring that employees feel their confidentiality will be respected in practice and not just on paper.

**De Beers**

De Beers provides HAART to workers and their partners as part of their comprehensive Wellness Programmes. Making treatment available has increased the uptake of VCT but misconceptions about HIV/AIDS and fears about the company's motives in offering testing initially prevented the expansion of the programme. Workers receiving HAART also had concerns that complying with the rigorous treatment schedule would make it obvious to their colleagues that they were HIV positive. This interfered with effective treatment, and has raised the spectre of growing drug resistance due to non-compliance. To offset these concerns, the company made confidentiality a priority.

#### Lessons learned

In order to deal with stigma and discrimination, confidentiality policies must be clear and widely communicated in appropriate language. Places of work should adapt facilities within their companies to allow for private discussions that help instil confidence.

Workplaces should also consider providing medical services through a network of practitioners outside the company, which can help workers feel that their privacy and confidentiality are assured. It can also help encourage compliance in those who have left the company due to ill health or retirement. Additional activities are needed to convince workers that they should inform their partner/s so that they, too, can be included in testing and treatment programmes.

### Voluntary Counselling and Testing (VCT): Increasing uptake of services

The ILO code is clear on the fact that HIV/AIDS screening should never be required of workers or job applicants, and should not be a condition for contract renewal. On the other hand, VCT is a key element in HIV prevention and is, as such, encouraged by many workplace programmes. In fact, testing is often the entry point for the provision of ARV and other therapies. Policy to protect workers from unwarranted testing is now widespread, but there is still much to learn from best practice in promoting VCT uptake.

**Eskom**

Eskom has a long-standing HIV/AIDS programme but found that stigma was exercising a major impact on the uptake of VCT. The company conducted a workplace prevalence study to understand workers’ responses and to plan adjustments to their use of peer educators and community outreach initiatives to allay fears.

#### Lessons learned

Plans to introduce counselling and testing programmes must be mindful of the fact that a positive diagnosis is an extremely painful realisation in that the person being tested has to acknowledge that he/she has a fatal infection. It may be helpful to link VCT to the provision of ARVs. This is a major incentive for testing, but accepting treatment may expose an individual and family members to discrimination and stigma.

This case study has shown that addressing workplace discrimination is not sufficient, as workers may fear stigma more than losing their jobs and may face secondary stigma and discrimination at home and in the community. Outreach work in communities will, therefore, enhance the success of testing at work.

Women face stigma even more than men do and thus efforts to increase VCT uptake should pay particular attention to addressing their specific concerns. Despite their complexity, VCT programmes are undoubtedly effective in terms of budgetary and other savings for enterprises.

### Treatment: Responding to opportunistic infections through partnership

Treatment through the provision of occupational health services at the workplace includes programmes to provide ART. The treatment of opportunistic infections such as TB and of STIs and other concurrent infections is also important. Similarly, the workplace can provide the setting for ensuring good nutrition which delays HIV progression, for delivering pain control and palliative care, and, when needed, for offering the psychosocial support that facilitates compliance with treatment. One of the objectives of activities under resource-constrained conditions is to ensure, as far as possible, that PLWHA are assisted in living positively.

**Pfizer – South Africa Alliance (Diflucan Partnership)**

Pfizer and the South African Ministry of Health have established a public-private partnership to address life-threatening, opportunistic fungal infections. The company donates treatment and supports training initiatives to ensure its proper use while the Ministry maintains the infrastructure for distribution and delivery. The experience has provided valuable experience of partnership and about treating conditions linked to HIV/AIDS.

#### Lessons learned

Access to drugs and the most up-to-date therapy is crucial to initiatives to treat opportunistic infections. Treatment is most effective when supported by a package of measures including advice on healthy living and nutrition. Workers often go to the health service as a result of opportunistic infections, and the contact established can usefully be exploited to address wider issues including VCT, the risk to family members and others, and prevention. When workers are not HIV positive, treating other infections can help reduce their risk of contracting HIV.

Training of health professionals in STI treatment should address treatment regimes and how to handle wider HIV/AIDS issues. This case study has shown that public-private partnerships can benefit from a clear agreement on the responsibilities of each stakeholder, contingency plans to cover potential problems and regular meetings to update and adjust programmes.

### Managing the transition from formal to informal

Most informal workers have little voice and very little control over working conditions. They often have to combine a mixture of types of work and women are particularly vulnerable in this regard. Many women are heads of households living in poverty, and are unlikely to insist on safe sex. This project attempts to link formal workplaces to informal ones and to government initiatives.

**Placer Dome Western Areas Joint Venture**

The Placer Dome Western Areas Joint Venture workplace programme for the South Deep Gold Mine originally sought to help staff who lost their jobs when the mine was no longer profitable. It helps staff as they become increasingly unwell from AIDS to take on other economic activities. The initiative follows on from comprehensive efforts at prevention, care and accommodation. The intention is to help them find other, less arduous means of income-generation so that they can make a dignified transition from work. It provides training, business planning and loans to help ‘retrenched’ or ‘medically repatriated individuals’ to find more suitable employment or to start up small, entrepreneurial or income-generating activities whether these be in the formal or informal sector.

#### Lessons learned

The initiative of managing the transition from a formal workplace programme to an informal one is made particularly effective if employees who are too ill to benefit from the programme are able to nominate a relative to take up the training and so ensure that the family has some income-generating capacity. Paying attendance allowances makes it possible for individuals to participate in the initial training and counselling.

Integrating a home-based care project (and the provision of monthly medical kits) with efforts to support income-generation helps ex-employees with AIDS to remain economically active, and their families to remain economically viable even after the death of the ex-worker. It is also essential to involve local leaders and traditional healers to ensure acceptance of the programme. This case study has shown that building a consortium (of eight mining companies) and using a per capita funding model can help fund innovative initiatives and attract external and government partners, but it does take time for partnerships to establish effective ways of working.

### The community: Treatment for STIs and social outreach

Instead of directly focusing on the prevention of HIV, this strategy focuses on the prevention of HIV transmission through the treatment of sexually transmitted infections amongst sex workers in the local community. STIs are known to put people at increased risk of getting infected with HIV.

**The Lesedi project**

The Lesedi project is sponsored by Harmony Gold Mine and extends services to sex workers to encourage prevention and to break the cycle of HIV infection. It provides sex workers with clinics and peer educators to promote condom use, and works with unions to encourage acceptance of condom use amongst workers. There is presumptive treatment of STIs, with antibiotics prescribed without establishing that individuals are infected, as prevalence is high and testing is difficult. The implications of this practice are being monitored.

#### Lessons learned

This community-oriented case study has shown that consultation and leadership are key and must include unions throughout. It is essential to monitor approaches that provide antibiotics as a rule of thumb, for they may lead to resistance and ultimately undermine condom use by creating a false sense of security in sex workers about HIV infection. Nursing practitioners are central to the treatment of STIs and should be trained on prescription practices.

The health departments should be involved consistently, not least because they can help in removing constraints on nursing practitioners but also because they have a crucial role to play in monitoring and evaluating the chosen strategy. Initiatives to address health issues are more effective when integrated with social upliftment schemes that include micro-finance schemes for alternatives to sex work.

### Situation analysis: Understanding what is needed

Data collection and situation analysis are vital as ways of ascertaining practical needs, and of identifying the correct stakeholders for involvement in designing responses to those needs. Large organisations are well placed to conduct HIV prevalence surveys (and need them because of the complexity of the multinational working environment), although they may be disproportionately expensive for small and medium enterprises (SMEs).

**DaimlerChrysler South Africa (DCSA)**

DCSA initiated a prevention and care programme in 1991, but it was not effective and infection rates and costs to the company continued to rise. A baseline survey of Knowledge, Attitudes, Perceptions and Behaviours (KAPB) found that people knew little about HIV infection or the services provided. This data helped in designing the response, including peer education, HAART provision, reasonable accommodation and guaranteed social security benefits, with evaluation built into the project. The revised approach was both more successful and more cost-effective, providing better protection for the business as well as opportunities to link with contractors in the informal sector and with the community.

#### Lessons learned

Situational analyses show that survey work can help identify why service uptake does not meet expectations. In this case, the problem lay in fears about confidentiality and insufficient marketing of the help provided.

A KAPB survey, together with union involvement and top-level support, guides detailed responses on how best to design and communicate features related to confidentiality and provides a baseline for monitoring.

Stigma is a major factor in discouraging VCT and seeking care, and needs to be better understood if information, education and communication campaigns are to combat it effectively. It is essential to set specific targets for VCT, TB cure and STI recurrence for monitoring performance and for keeping costs under control.

### Monitoring and feedback

No initiative can be designed without flaws or without room for improvement, and no situation will remain static over time. Therefore, monitoring and feedback are crucial if adjustments and adaptations are to be made which will allow best practice to move beyond the innovation stage. Lessons of best practice need to be documented if this experience is to be available to others who are contemplating workplace activities. It is thus essential that programmes have defined and assessable objectives and that systems for monitoring these objectives are established from inception.

**The Department of public Service and Administration (DPSA)**

The public service has about 140 departments and 1.1 million members of staff. It recognises the importance of HIV policy, particularly in terms of the impact of staff losses on the department's ability to carry out its core functions as demands increase with the epidemic. There has been an attempt to identify core service areas, scarce skills and key posts, and to protect these by means of priority programmes. The policy development phase has opted for the creation of a set of minimum mandatory requirements to be incorporated into Public Service Regulations. Each of these requirements (including education, links with health promotion, and establishment of an HIV/AIDS committee per department, non-discrimination, testing and confidentiality) is linked to a monitoring obligation and a set of annual reports.

#### Lessons learned

The Department of Public Service and Administration (DPSA) has learned from this case study that baseline information is needed before monitoring methods can be put in place. It is also essential to set tangible targets for deliverables and monitoring progress against those targets. This is a highly effective management tool that prompts action and highlights shortcomings.

It is helpful to develop a straightforward set of indicators which can be easily updated and can provide day-to-day management information (although more detailed annual reviews are also useful and worthwhile). Evaluation should involve all stakeholders, including unions and workers’ representatives, and should combine quantitative and qualitative elements if it is to be credible. Feedback must be clear and transparent if it is to inspire change. Governments can encourage monitoring, evaluation and feedback through example and by making their support of (or partnership in) any project conditional on proper monitoring and reporting measures being put in place.

### Conclusion

It is encouraging that over one-third of all best practice workplace HIV interventions identified by the ILO were from South Africa. A majority of these policies and programmes focused on prevention. A call for greater corporate involvement in the response to HIV/AIDS has spurred several companies in South Africa on to expand workplace programmes to provide ARV treatment for their employees.

Business leaders in South Africa increasingly recognise that providing care and treatment for HIV-infected employees is as much about risk management as it is about corporate responsibility. Improving care and treatment for HIV-infected employees goes beyond altruism; it also makes good financial sense. Many companies that are now implementing HIV/AIDS programmes conducted risk assessments that indicated that the provision of ARV treatment would have significant cost benefits - reducing absenteeism, hospitalisation, and death, and thus lowering the expenses associated with recruiting and training new employees.

An encouraging number of interventions are providing links beyond the workplace while some have focused on policy and legal frameworks. The private sector has been able to provide better quality services to workers and their dependants than the government. Collaborations between the private and public sectors can also expand the scope of HIV/AIDS workplace programmes, and many companies have formed partnerships with the government, international donors and NGOs.

It is disappointing that only two workplace HIV/AIDS programmes focused on evidence obtained from KAPB and risk assessment surveys to develop their interventions. Companies should conduct risk assessments before initiating prevention programmes and before providing ARV treatment for their workers. There is a need to expand innovative workplace HIV interventions to small-, medium- and micro-enterprises.
